# Perspective and consideration in the application of personalized reference intervals based on biological variation: a four-month observation of a woman with SARS-CoV-2 reinfection

**DOI:** 10.11613/BM.2025.030901

**Published:** 2025-08-15

**Authors:** Gaofeng Hu, Lei Xu, Kai Guo, Chenbin Li

**Affiliations:** 1 National Center for Clinical Laboratories, Institute of Geriatric Medicine, Chinese Academy of Medical Sciences, Beijing Hospital/National Center of Gerontology, Beijing, PR China; 2 Department of Clinical Laboratory, Peking University First Hospital, Beijing, PR China; 3 Beijing Key Laboratory for Genetics of Birth Defects, Beijing Pediatric Research Institute, Genetics and Birth Defects Control Center, National Center for Children’s Health, MOE Key Laboratory of Major Diseases in Children, Beijing Children’s Hospital, Capital Medical University, Beijing, P. R. China

**Keywords:** biological variation, hematology, personalized reference intervals, biological variation, reference change value, reference intervals

## Abstract

This study aimed to investigate potential benefit of personalized reference intervals (prRIs) by conducting a four-month observation of a woman with SARS-CoV-2 reinfection. Two types of prRIs were calculated: one derived from the population biological variation (BV) data provided by the European Federation of Clinical Chemistry and Laboratory Medicine (EFLM) biological variation database (prRIs__pop._), and the other derived from individual variation data (prRIs__ind._). These were subsequently compared. A total of 110 test results encompassing complete blood count (CBC) and leukocyte differential counts from the case were assessed according to the limits of prRIs__pop._, reference change values (RCVs__pop._) and the population-based reference intervals (popRIs). In instances where limited historical health data are available (N ≤ 3), the application of prRIs__pop._ was recommended over prRIs__ind_. The prRIs__pop._ and RCVs__pop._ identified a greater number of potential clinical pathological change compared to popRIs (the ratio of potential abnormal values to total test values: prRIs__pop._ 22/110, RCVs__pop._ 25/110, popRIs 2/110, respectively). The findings suggest that the use of prRIs can be advantageous in clinical settings and is worthy of broader adoption. However, it is essential to choose an appropriate calculation method tailored to the specific clinical context.

## Graphical abstract

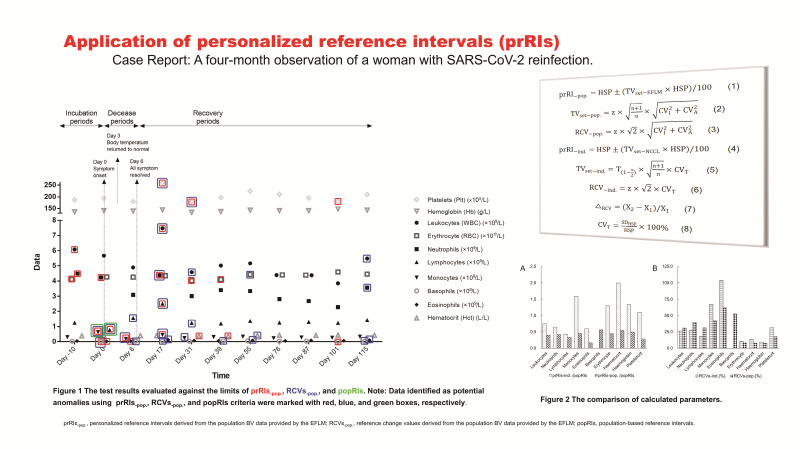

## Highlights

• Personalized reference intervals offer substantial clinical value and utility

• Recommended to use published biological variation data for the calculation

• The reference change value can be effectively employed as a supplementary method

## Introduction

The interpretation of laboratory data relies on reliable reference data for comparison, which are typically derived from population-based data statistics but applied to individuals. However, using population-based reference intervals to interpret test results for individual patients presents significant limitations in diagnosing, monitoring, and treating single individuals. To address this issue, Coşkun and colleagues developed a novel method for calculating personalized reference intervals (prRIs) based on biological variation (BV) (*1*-*3*). This approach allows patients’ test results to be compared against their own individualized reference intervals, offering potential benefits in diagnosis and patient follow-up. Despite its promise, the adoption of individualized reference intervals has not yet received adequate attention in the field. In this study, we present a representative case study conducted over a four-month period to assess the advantages of utilizing prRIs for monitoring patients in real-world clinical practice.

## Case presentation

### Patient

A 33-year-old woman presented with fever (38 °C), dry cough, sore throat, and myalgia. She tested positive for SARS-CoV-2 antigen using two different kits on 2 consecutive days. After taking antipyretic drugs, her body temperature returned to normal by Day 3, and her remaining symptoms resolved within one week. Prior to this episode, she had received three doses of COVID-19 vaccine (Sinopharm Beijing) and had experienced her first SARS-CoV-2 infection 9 months earlier. The study was conducted in compliance with the ethical guidelines established by the Institutional Review Board of Beijing Hospital (Approval Letter No. 2023BJYYEC-194-01). Written informed consent was obtained from the case presented in this report.

### Laboratory work-up

We collected complete blood count (CBC) results, including leukocytes, erythrocyte, hematocrit, hemoglobin and platelets, as well as and leukocyte differential counts, including neutrophils, lymphocytes, monocytes, eosinophils and basophils, at 11 time points from Day - 10 to Day 115 (with Day 0 was defined as the day of symptom onset) before and after the SARS-CoV-2 reinfection. All laboratory tests were conducted at the National Center for Clinical Laboratories (NCCL), Beijing Hospital.

The population-based reference intervals (popRIs) were derived from the China national health standard WS/T 405 (available at http://www.nhc.gov.cn/ewebeditor/uploadfile/2013/01/20130109171100186.pdf). Personalized reference intervals (prRIs) and reference change values (RCVs) were calculated using the equations proposed by Coşkun and colleagues (*1*, *2*). The homeostatic set point (HSP) was estimated based on results from her three previous physical examinations conducted between 3 years and 5 months previously (N = 3). Due to the limited number of data points available for HSP calculation, two distinct methods were employed to determine the prRIs and RCVs: one derived from population BV data (prRIs__pop._ and RCVs__pop._), and the derived from individual variation data (prRIs__ind._ and RCVs__ind._). The formulas for these calculation (Equations (Eq.) 1-8) are provided below.


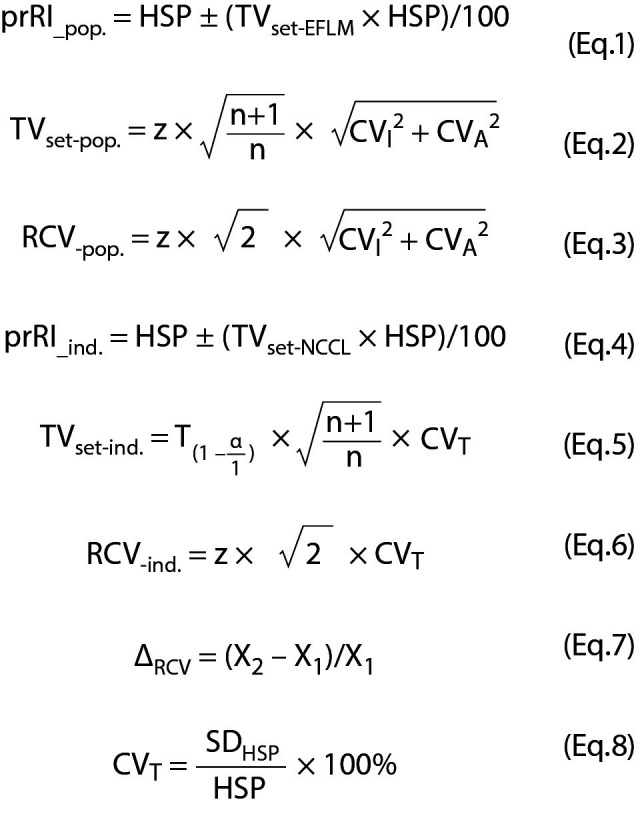


Note: z = 1.96 for 95% probability; is the t-table value with n−1 freedom (two-side); UL, upper limit; LL, lower limit; N/A, not applicable; TV_set_, the total variation around the true homeostatic set point.

The within-subject biological variation (CV_I_) and between-subject biological variation (CV_G_) data were sourced from European Federation of Clinical Chemistry and Laboratory Medicine (EFLM) BV database (*4*). The analytical variation (CV_A_) data were obtained from NCCL. The “total variation” of the results (CV_T_) is the combination of within-person variation (CV_P_) and CV_A_ and calculated from the HSP data of the case with SARS-CoV-2 reinfection (*3*).

**Figure 1:**
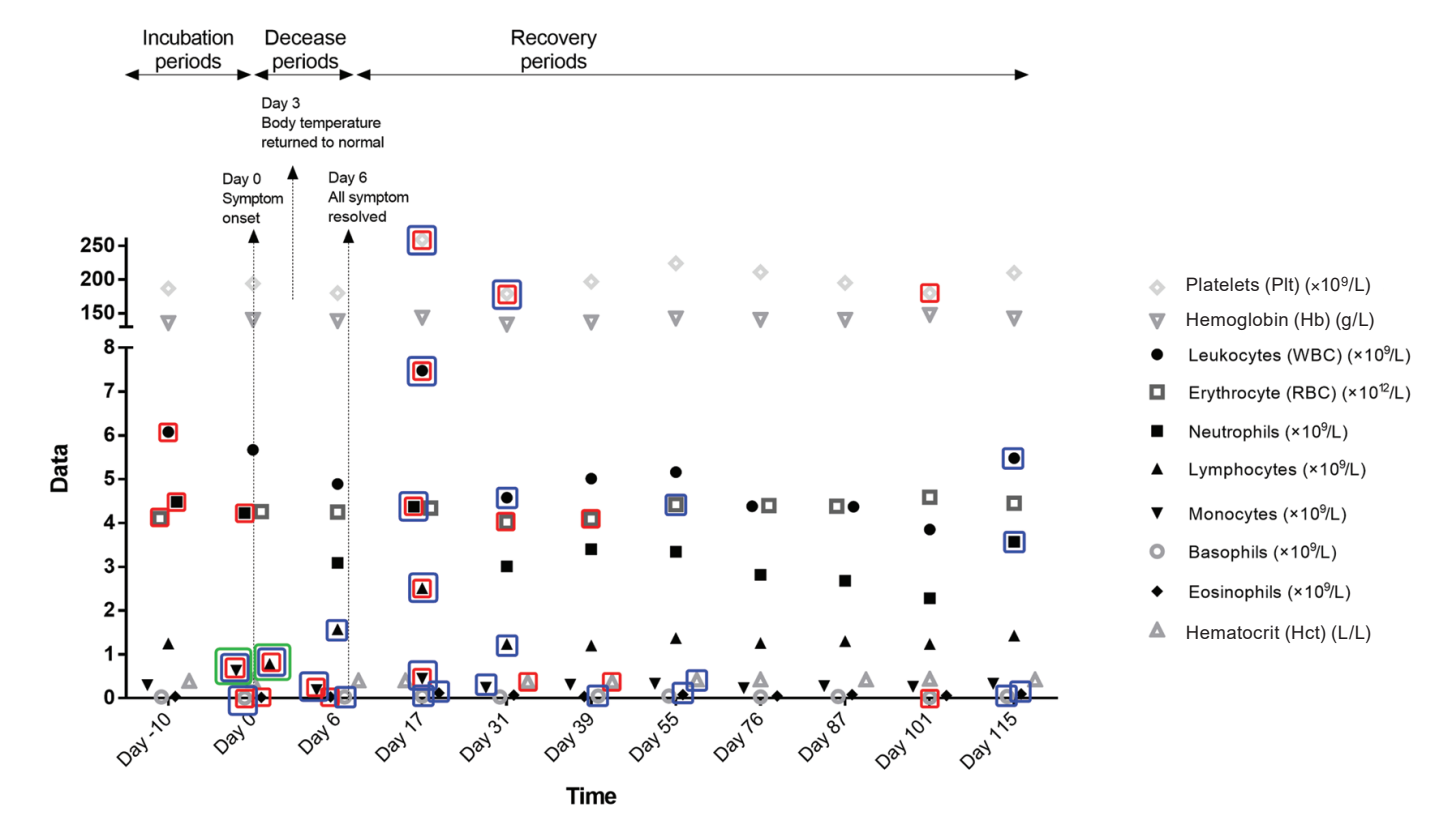
The test results evaluated against the limits of prRIs**_**_pop._, RCVs**_**_pop._ and popRIs. Data identified as potential anomalies using, prRIs**_**_pop._, RCVs**_**_pop._ and popRIs criteria were marked with red, blue, and green boxes, respectively. prRIs**_**_pop._ - personalized reference intervals derived from the population biological variation data provided by the EFLM. RCVs**_**_pop._ - reference change values derived from the population biological variation data provided by the EFLM. popRIs - population-based reference intervals.

The calculated parameters are summarized in Table 1. The index of individuality (II), defined as the ratio of CV_I_ to CV_G_ based on data form the EFLM BV database, ranged from 0.24 to 0.65 (*1*, *4*). The range of the prRIs__pop._ (upper limit - lower limit) for all 10 tested parameters were narrower compared to the popRIs. However, the prRIs__ind._ for only 5 of the tested parameters (leukocytes, neutrophils, lymphocytes, eosinophils, and basophils) were narrower than the popRIs (Table 1 and Figure 1-A). Additionally, the RCVs__pop._ of 6 of the tested parameters were lower than RCVs__ind._ (Table 1 and Figure 1-B).

**Table 1:** Summary of calculated parameters for complete blood count and leukocyte differential count

	**HSP**	**CV_I_ (%)**	**CV_A_ (%)**	**CV_T_ (%)**	**II**	**popRIs**	**prRIs__pop._**	**RCVs__pop._ (%)**	**prRIs__ind._**	**RCVs__ind._ (%)**
Leukocytes (x 10^9^/L)	4.8 ± 0.5	11.10	1.26	9.45	0.65	3.5 ~ 9.5	3.7 ~ 6.0	30.96	2.5 ~ 7.0	26.20
Neutrophils (x 10^9^/L)	3.0 ± 0.3	14.10	1.46	9.83	0.58	1.8 ~ 6.3	2.0 ~ 3.9	39.29	1.5 ~ 4.4	27.25
Lymphocytes (x 10^9^/L)	1.4 ± 0.1	10.80	3.55	6.62	0.48	1.1 ~ 3.2	1.0 ~ 1.7	31.51	0.9 ~ 1.8	18.34
Monocytes (x 10^9^/L)	0.3 ± 0.1	13.30	7.08	24.24	0.60	0.1 ~ 0.6	0.2 ~ 0.4	41.77	- 0.1 ~ 0.7	67.20
Eosinophils (x 10^9^/L)	0.1 ± 0.0	15.00	16.70	37.50	0.24	0.02 ~ 0.52	0.04 ~ 0.12	62.23	- 0.1 ~ 0.2	103.94
Basophils (x 10^9^/L)	0.04 ± 0.0	12.40	14.35	0.00	0.44	0 ~ 0.06	0.02 ~ 0.06	52.56	0.0 ~ 0.04	0.00
Erythrocytes (x 10^12^/L)	4.45 ± 0.17	2.80	0.67	3.82	0.40	3.8 ~ 5.1	4.16 ~ 4.74	7.98	3.61 ~ 5.30	10.59
Hematocrit (L/L)	0.420 ± 0.020	2.80	0.54	4.76	0.50	0.35 ~ 0.45	0.390 ~ 0.450	7.90	0.320 ~ 0.520	13.20
Hemoglobin (g/L)	141 ± 5	2.70	0.25	3.36	0.44	115 ~ 150	132 ~ 149	7.52	117 ~ 164	9.32
Platelets (x 10^9^/L)	217 ± 25	6.40	0.17	11.43	0.47	125 ~ 350	185 ~ 249	18.06	94 ~ 341	31.69
HSP - homeostatic set point presented as mean and standard deviation. CV_I_ – within-subject biological variation. CV_A_ – analytical variation. CV_T_ – total variation. II - index of individuality, *i.e.* CV_I_/CV_G_. popRIs - population-based reference intervals. prRIs**_**_pop._ – personalized reference intervals derived from population biological variation data. RCVs**_**_pop._ - reference change values derived from population biological variation data. prRIs**_**_ind._ - personalized reference intervals derived from individual variation data. RCVs**_**_ind._ - reference change values derived from individual variation data. Reference intervals are presented as lower to upper limit.										

**Figure 2:**
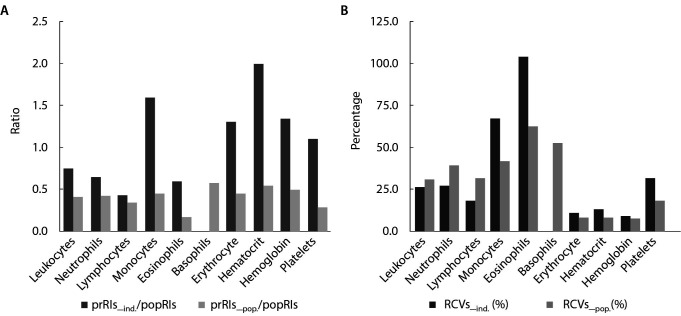
The comparison of calculated parameters. prRIs**_**_pop._ - personalized reference intervals derived from the population biological variation data provided by the EFLM. RCVs**_**_pop._ - reference change values derived from the population biological variation data provided by the EFLM. popRIs - population-based reference intervals. RCVs**_**_ind._ - reference change values derived from individual variation data. RCVs**_**_pop._ - reference change values derived from population biological variation data.

A total of 110 test results were evaluated against the limits of prRIs__pop._, RCVs__pop._ and popRIs, as detailed in Figure 2 and Supplementary Table 1. The analysis revealed that prRIs__pop._ and RCVs__pop._ identified a great number of potential clinical pathological change compared to popRIs. Specifically, the ratios of potential abnormal values to the total number of test values were as follows: prRIs__pop._ 22/110, RCVs__pop._ 25/110, and popRIs 2/110 (Figure 2 and Supplementary Table 1).

## Discussion

In the pursuit of precision medicine, the development of the new prRIs algorithm based on the homeostatic model represents a significant advancement in the field of clinical laboratory medicine. Mathematically, the upper and lower limits of prRIs are determined by HSP and the total variation around the true HSP (TV_set_) (*1*-*3*). However, one of the primary challenges in calculating prRIs is the availability of reliable and sufficient steady-state data (*5*, *6*). The critical factor lies in accurately identifying steady-state condition, which are not synonymous with healthy condition. In chronic diseases such as diabetes, asymptomatic hyperuricemia, and malnutrition-related anemia, which may have been present for years before diagnosis, previous test results may not necessarily reflect the true HSP. Therefore, it is essential to implemented both prRIs and popRIs in tandem. Additionally, outlier and trend analysis are indispensable tools in this process. However, we have reservations about automatically excluding data that fall outside the popRIs limits when calculating prRIs. In some cases, an individual may be in a steady-state condition, yet their test results may lie outside the popRIs limits. We recommended using RCVs or other robust method to determine whether such data should be excluded. RCVs, calculated based on 2 consecutive results from an individual, are independent of analyte’s HSP. Nevertheless, it is important to note that a single test result cannot definitively indicate whether a recovery from a pathological state or a new pathological change has occurred. When reliable HSP data are available, prRIs offer the highest sensitivity for interpreting test results and are most conductive to facilitating prompt clinical decision-making among the three types of intervals (prRIs, RCVs, and popRIs). In summary, the clinical application of prRIs requires a long-term awareness of health management and imposes higher quality control requirements for both the pre-analytical and post-analytical phases of the testing process. This underlines the importance of close collaboration between clinicians and laboratory professionals. This approach ensures the accurate and effective use of prRIs in advancing personalized patient care.

The estimation of TV_set_ can be performed using two equations based on CV_I_ or CV_P_, which rely on either the normal distribution or t-distribution (see Eq. 2 and 5). The EFLM Biological Variation Database provides freely accessible CV_I_ estimates derived from global published BV studies and meta-analysis (*4*). However, for CBC and leukocyte differential counts, the specimens are analyzed immediately after collection due to their instability. Consequently, the calculated CV_I_ data may be confounded by between-run analytical variation (*7*). Although newer statistical methods, such as CV-ANOVA, the Bayesian approach, and big data mining, offer more scientific and efficient tools for reliable BV estimation, they do not fundamentally resolve this issue (*8*). Coşkun and colleagues advocate for the use of CV_P_ over CV_I_ when deriving prRIs, as CV_P_ more accurately reflects the BV of a single individual, whereas CV_I_ represents the “average” BV of a population (*9*). In this study, we estimated two types of prRIs: prRIs__pop._ and prRIs__ind._. When historical health data are limited, such as with a sample size of N = 3, the range of prRIs__ind._ can become excessively wide, potentially exceeding that of popRI. This diminished the advantages of using prRIs. As recommended by Coşkun, at least 5 previous test results from a steady-state condition are needed. The widespread use of laboratory information system has made more data accessible to laboratory professionals. While, accumulating sufficient data to calculate CV_P_ for all patients is both time-consuming and often impractical for most laboratories. Therefore, it may be more feasible to calculate and utilize prRIs__pop._ and RCVs__pop._ in clinical practice. Additionally, the CV_I_ estimates of CBC and leukocyte differential counts in EFLM BV database are derived from meta-analysis. Given the rapid advancements and innovation in detection technology, we recommend prioritizing CV_I_ estimates from studies with higher Biological Variation Data Critical Appraisal Checklist (BIVAC) grades over meta-analysis results. This approach ensures more accurate BV estimates tailored to specific populations and detection technologies.

Most clinical laboratories rely on published CV_I_ data and their own laboratory’s CV_A_ data to calculate prRIs. It is widely accepted that CV_A_ should be less than or equal to half of CV_I_ (CV_A_ ≤ 0.5CV_I_) when estimating BV, and measurands with CV_I_ > 30% are generally considered unsuitable for prRIs calculations (*3*, *7*). This implies that the CV_A_ of a measurand should ideally be less than 15%. However, due to limitations in measurement methods, the CV_A_ for eosinophils and basophils in our laboratory were 16.7% and 14.4%, respectively. In such case, RCVs may be a more suitable alternative. In fact, each laboratory should establish its own analytical performance specifications (APS) for measurands. Importantly, prRIs are not only individualized but also laboratory-specific, which adds complexity to the standardization and harmonization efforts in laboratory medicine.

The index of individuality (II) is commonly used to evaluate the applicability popRIs (*1*). Two equations are typically used for II calculation: II = CV_I_/ CV_G_ and (*10*). While there is no- consensus on which formula is preferable, the former is more frequently used. The II values for CBC and leukocyte differential counts, calculated using data from the EFLM ranged from 0.24 to 0.65 (4). This indicates that conventional popRIs have very limited utility in identifying abnormal results for a specific individual (*7*). Ideally, clinical laboratories should reansition to using prRIs rather than popRIs. The findings in this case revealed that the popRIs criterion could only identify a limited number of potential outliers, whereas the prRIs__pop._ and RCVs__pop._ criteria demonstrate broader applicability by identifying more potential abnormal values across different disease phases including the incubation and recovery periods. While these results are derived from a single case observation, they imply that the employment of more sensitive criteria (prRIs__pop._ and RCVs__pop._) could enhance the detection of clinically significant variations in future cohort studies and may help uncover additional patterns in disease progression. Nevertheless, the preponderance of extant evidence corroborates the indispensability of population-based reference intervals (popRIs) for parameters exhibiting an index of individuality greater than 0.6. Consequently, emphasis is placed on the necessity of selecting popRIs, prRIs, or a combination, for the interpretation of results, with this selection being based on each parameter’s intrinsic biological characteristics.

In summary, this case study demonstrates the significant potential of population biological variation-based prRIs in improving clinical decision-making. The limitations of this study are the data from only one woman with SARS-CoV-2 reinfection and the serial measurements of blood cells, but this study provides unique contributions: (*1*) a comprehensively documented longitudinal dataset spanning the entire disease course (incubation, disease, and recovery phases); and (*2*) rigorously standardized laboratory procedures ensuring exceptional comparability across all tests, including the three HSP measurements. Despite the considerable challenges that still need to be overcome for the widespread implementation of prRIs - and full integration into routine clinical practice remains a long-term goal - the ongoing discussion and critical evaluation of the utility and importance of laboratory measurements remain valuable.

## Data Availability

The data generated and analyzed in the presented study are available from the corresponding author on request.

## References

[1] Coşkun A Sandberg S Unsal I Cavusoglu C Serteser M Kilercik M Personalized Reference Intervals in Laboratory Medicine: A New Model Based on Within-Subject Biological Variation. Clin Chem. 2021;67:374–84. 10.1093/clinchem/hvaa233 33188412

[2] Coşkun A Sandberg S Unsal I Yavuz FG Cavusoglu C Serteser M Personalized reference intervals - statistical approaches and considerations. Clin Chem Lab Med. 2021;60:629–35. 10.1515/cclm-2021-1066 34894385

[3] Coşkun A Sandberg S Unsal I Cavusoglu C Serteser M Kilercik M Personalized and Population-Based Reference Intervals for 48 Common Clinical Chemistry and Hematology Measurands: A Comparative Study. Clin Chem. 2023;69:1009–30. 10.1093/clinchem/hvad113 37525518

[4] Aarsand AK, Fernandez-Calle P, Webster C, Coşkun A, Gonzales-Lao E, Diaz-Garzon J, et al. The EFLM Biological Variation Database. Available at: https://biologicalvariation.eu/. Accessed: August 23, 2024.

[5] Coşkun A Sandberg S Unsal I Cavusoglu C Serteser M Kilercik M Personalized reference intervals: Using estimates of within-subject or within-person biological variation requires different statistical approaches. Clin Chim Acta. 2022;524:201–2. 10.1016/j.cca.2021.10.034 34743811

[6] Carobene A Banfi G Locatelli M Vidali M. Personalized reference intervals: From the statistical significance to the clinical usefulness. Clin Chim Acta. 2022;524:203–4. 10.1016/j.cca.2021.10.036 34743810

[7] Fraser CG Harris EK. Generation and application of data on biological variation in clinical chemistry. Crit Rev Clin Lab Sci. 1989;27:409–37. 10.3109/10408368909106595 2679660

[8] Sandberg S Carobene A Bartlett B Coşkun A Fernandez-Calle P Jonker N Biological variation: recent development and future challenges. Clin Chem Lab Med. 2022;61:741–50. 10.1515/cclm-2022-1255 36537071

[9] Coşkun A Sandberg S Unsal I Serteser M Aarsand AK. Personalized reference intervals: from theory to practice. Crit Rev Clin Lab Sci. 2022;59:501–16. 10.1080/10408363.2022.2070905 35579539

[10] Plebani M Padoan A Lippi G. Biological variation: back to basics. Clin Chem Lab Med. 2015;53:155–6. 10.1515/cclm-2014-1182 25568984

